# Spatial and socio-demographic determinants of contraceptive use in the Upper East region of Ghana

**DOI:** 10.1186/s12978-015-0017-8

**Published:** 2015-04-02

**Authors:** Fabian Sebastian Achana, Ayaga A Bawah, Elizabeth F Jackson, Paul Welaga, Timothy Awine, Eric Asuo-Mante, Abraham Oduro, John Koku Awoonor-Williams, James F Phillips

**Affiliations:** Navrongo Health Research Centre, P. O. Box 114, Navrongo, Upper East Region Ghana; Department of Population and Family Health, Columbia University Mailman School of Public Health, 60 Haven Avenue, B2, 10032 New York, NY USA; Regional Health Directorate, Ghana Health Service PMB, Upper East Region, Bolgatanga, Ghana; Swiss Tropical and Public Health Institute, Socinstrasse 57, 4002 Basel, Switzerland; Switzerland University of Basel, Peterplatz, 4003 Basel, Switzerland

**Keywords:** Spatial, Social determinants, Contraceptive use, Family planning, Primary care, Women, Ghana

## Abstract

**Background:**

This paper presents results of baseline data on the determinants of contraceptive use in 7 districts in northern Ghana where there is an ongoing integrated primary health care systems strengthening projectknown as the Ghana Essential Health Intervention Project (GEHIP).

**Methods:**

We used a household survey data conducted within 66 randomly sampled census enumeration areas in seven rural districts of the Upper East Region of northern Ghana where health systems strengthening interventions are currently ongoing in three of the districts with four of the districts serving as comparison districts. This survey was conducted prior to the introduction of interventions. Data was collected on various indices included geographic information systems (GIS) and contraceptive use. The data was analyzed using survey design techniques that accounts for correct variance estimation. Categorical variables were summarized as proportions and associations between these variables and contraceptive use tested using Chi-square test. Uni-variable and multivariable logistic regression techniques were used to assess the effects of the selected covariates on contraceptive use. All tests were deemed to be statistically significant at 5% level statistical significance.

**Results:**

Results show that contraceptive use is generally low (about 13 per cent) and use is nearly evenly for spacing and stopping purposes. Factors associated with the use of contraceptives include exposure to integrated primary healthcare services, the level of education, and socioeconomic status, couple fertility preference, marital status, and parity. For instance, the odds of contraceptive use among 15–45 year old women who live 2 km or more from a CHPS compound is 0.74 compared to women who live less than 2 km from a CHPS compound (p-value = 0.035).

**Conclusion:**

The findings suggest that rapid scale up of the Community based Health Planning and Services (CHPS) program accompanied with improved door-to-door health services would kindle uptake of modern contraceptive use, reduce unwanted pregnancies and hasten the attainment of MDG 4 & 5 in Ghana.

## Background

It is widely assumed that improving access to family planning services will reduce the social, logistical, and economic cost of contraceptive practice [1]. This paper presents an analysis of data collected in a situation that challenges this assumption. Despite considerable progress with extending the coverage of community-based primary health care in Ghana’s Upper East Region (UER) [2], and policies that integrate comprehensive family planning into community-based primary health care services [3,4] contraceptive prevalence in the region remains low relative to levels in the regions of southern Ghana [5]. While some researchers have attributed the relatively low prevalence in the region to lack of access to contraceptive services [6], others have emphasized the role of social and cultural constraints to contraceptive use throughout northern Ghana [7,8]. Constraining factors arising from interaction of poor service quality, lack of knowledge, fear of side effects, and social and familial disapproval have also been documented [9]. Some authors have also attributed the limited impact of family planning programs in Ghana and much of sub-Saharan Africa to the neglect of men as equal targets [10-12].

This paper reports results from a household cluster survey that was conducted by the Ghana Essential Health Intervention Programme (GEHIP) in 2011 [13]. The survey was conducted in 66 randomly selected census enumeration areas in the seven GEHIP districts. GEHIP is a health system’s strengthening initiative that is aimed at providing supportive structures to district health delivery programs to improve on child health and survival. GEHIP is being implemented in seven districts, three of which are implementing various health systems interventions with four of the districts serving as comparison districts. It is anchored on Ghana’s primary healthcare programme – Community-based Health Planning and Services (CHPS) programme which is aims to deliver health services to rural communities throughout the country. Data from the survey permits an appraisal of spatial and socials determinants of contraceptive use in the region.

This paper assesses the spatial and socio-demographic determinants of contraceptive use in Upper East region. The overarching aim is to assess the effects of proximity to service points relative to social determinants on contraceptive use. If the expansion of access to CHPS facilities is having its desired effect on improving access, we would expect to find that distance to nearest health facility inversely related to contraceptive use. Wherever CHPS health posts are constructed, convenient access to family planning is expected to offset the detrimental effects of remoteness of sub-district health centers and hospitals.

## Methods

### The setting

The Upper East region (UER) is located in the north-eastern corner of Ghana and bordered by Burkina Faso to the north and Togo to the east, to the west by the Sissala District in the Upper West region and to the south by the West-Mamprusi District in the Northern Region of Ghana. It lies between longitude 0° and 1° west, and latitudes 10° 30′N and 11°N. The climate is characterized by one erratic rainy season from May/June to September/October and a long spell of dry season spanning November to mid-February, characterized by cold, dry and dusty harmattan winds.

The Upper East region is made up of 10 administrative districts but at the time of the survey, there were only 9 districts. However, in 2012, one of the districts was split into two making a total of 10 districts now. The survey was conducted in seven districts. Considered as the poorest in Ghana, the region inhabits 4.2% of Ghana’s estimated 25 million people, predominantly rural (79%) and considered to be one of Ghana’s poorest regions. [14]. Agriculture is the main economic activity of the people dominated by the cultivation of cereals, millet, sorghum, rice and the rearing of animals and domestic birds.

Illiteracy is quite pronounced in the region, with as high as 44.5 percent of the people reported as never been to school. Early marriage and childbearing and their associated adverse consequences are common. The total fertility rate (TFR) in the region is 4.1 compared to national TFR of 2.5 in Greater Accra; the national capital [5].

The region has one regional referral hospital and five district hospitals supported by several Community-based Health Planning and Services (CHPS) service points that provide critical primary care services to people in rural localities. CHPS is Ghana’s flagship primary care program to deliver healthcare services to rural communities. By 2011 when the survey was conducted half of the communities in UER were covered by CHP, the highest in the country [15]. Private health providers mainly missionary health facilities contribute significantly to the provision of health care services in the region [16].

**Figure 1:**
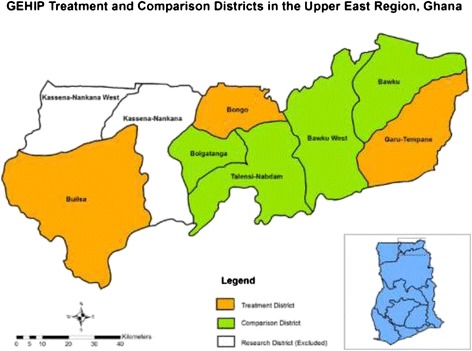
**Map of study area in the Upper East Region of Ghana.**

Figure 1 below is a map of the Upper East Region showing the GEHIP treatment and comparison districts as at the time of the survey. As Figure 1 shows, two research districts of the Navrongo Health Research Centre and an urban census enumeration area of the regional capital, Bolgatanga, were excluded in the baseline survey.

### Data and methods

Data for this paper come from a household cluster survey conducted in 66 randomly sampled census enumeration areas (EAs) in seven districts of the Upper East region of Ghana. The survey was a baseline survey implemented to collect various health and demographic indices prior to the introduction of the GEHIP programme. The survey was conducted during the months of April through August of 2011. The sampled EAs covered rural areas of the seven districts, three intervention and four non-intervention districts. The sampling was a two-staged procedure where the EAs were randomly selected from the 2010 population and housing census of Ghana enumeration areas (EAs) data base. The second stage involved a random sampling of households within the 66 EAs and women aged 15–49 years within those households interviewed. Overall, respondents were drawn from a total of 1,769 household across in the seven districts. Breakdown by district is as follows: Bawku East 329 households; Bawku West 193; Bolgatanga 208; Bongo 38; Builsa 280; Garu-Tempani 262 and Talensi-Nabdam 262.

The data collection instrument was adapted from the Ghana Demographic and Health Survey instrument [17] and modified to captured additional information that would allow for measuring the impact of the GEHIP programme. Prior to the data collection, the survey questionnaire was pre-tested and revised to ensure its appropriateness, validity and reliability.

Modern contraceptive use was defined using women’s responses to two survey questions: (1) Are you or your husband/partner currently doing something or using any method to delay or avoid getting pregnant?; and (2) Which method are you currently using? Respondents were classified as modern contraceptive users if they indicated that they were currently using sterilization, intra-uterine device (IUD), injection, implant, pill, male or female condom or some other modern method. Otherwise, they were considered non-users of modern methods of contraception. The socioeconomic status variable (SES) or wealth index variable was generated using household assets and possessions.

Exposure to the integrated primary healthcare services, socioeconomic and demographic characteristics that were theorized to affect modern contraceptives method use were selected based on a review of the literature [18].

### Statistical analysis

Geo reference data of residence location and all health facilities within the study area was used to compute the distance to the nearest health facility. We modeled distance to the nearest health facility using quadratic splines in other to determine suitable points of inflections. One point of inflection at 2 km was identified and the distance to the nearest health facility was then categorized as less than or equal to 2 km or more than 2 km. The data was analyzed using survey design techniques that accounts for correct variance estimation due to clustering. Categorical variables were summarized as proportions and associations between these variables and contraceptive use tested using Chi-square test. Uni-variable and multivariable logistic regression techniques were used to assess the effect of each of the selected covariate on modern contraceptive use [19]. All tests were deemed to be statistically significant at 5% level statistical significance.

### Ethical approval

The Navrongo Health Research Centre Institutional Review Board (IRB) and the Ghana Health Service Ethical review Board reviewed and approved the GEHIP project. Informed consent was obtained from heads of households as well as individual participants before participation in the study. Participation was entirely voluntary and participants could discontinue an interview at any time they so wished. We also protected the privacy and confidentiality of participants during data collection as well as excluding all personal information in the data processing, analysis and publications.

## Results

Background characteristics of the survey respondents are presented in Table 1. A total of 6000 women eligible were sampled of which 5511 were successfully interviewed; yielding a successful response rate of (91.9%). The survey was done in predominantly rural parts of the region thus the high percentage (87 percent) of rural representation. Educational attainment is very low: over 60 percent of the respondents have not been to school, and only 5.1% have had secondary/tertiary education. The rest have either primary/junior high school (JHS) education. Majority of women (55.8 percent) are Christians. A small fraction of respondents (3.6%) reported no religious affiliation. In terms of occupation, the respondents are predominantly farmers or traders (80 percent). Only 1.4 percent works in the formal sector as public or civil servants while 19.1 percent are students.

**Table 1:** **Background characteristics and exposure to CHPS among women 15–49 years old by use of modern contraceptives in 7 districts in Upper East Region**

		**Modern contraceptive use**		
**Variable**	**N = 5511 n (%)**	**Use contraceptives**	**Does not use**	**P-value**
**Prevalence of contraceptive use**	**5511 (100)**	**715 (13.0)**	**4796 (87.0)**	<0.001
**Place of residence**				
Urban	716 (13.0)	101 (14.1)	615 (85.9)	0.334
Rural	4795 (87.0)	614 (12.8)	4181 (87.2)	
**Exposure to nearest health facility**				
<2 Kilometers	4796 (87.0)	501 (14.6)	2941(85.4)	<0.001
> = 2 Kilometers	715 (13.0)	214 (10.3)	1855 (89.7)	
**Level of education attained**				
None	3352 (60.8)	434 (12.9)	2918 (87.1)	<0.001
Primary/JHS	1877 (34.1)	221 (11.8)	1656 (88.2)	
Secondary/Tertiary	282 (5.1)	60 (21.3)	222 (78.7)	
**Age group**				
15-19	1211 (21.9)	68 (5.6)	1143 (94.4)	<0.001
20-34	2449 (44.5)	413 (16.8)	2036 (83.2)	
35-49	1851 (33.6)	234 (12.6)	1617 (87.4)	
**Religious affiliation**				
Christianity	3073 (55.8)	419 (13.6)	2654 (86.4)	0.180
Traditional	728 (13.2)	99 (13.6)	629 (86.4)	
Islam	1508 (27.4)	176 (11.7)	1332 (88.3)	
No Religion	202 (3.6)	21 (10.4)	181 (89.6)	
**Marital status**				<0.001
Married and in polygamous union	1374 (24.9)	162 (11.8)	1212 (88.2)	
Married and in a monogamous union	2272 (41.2)	410 (18.1)	1862 (81.9)	
Not married	1804 (32.7)	134 (7. 4)	1670 (92.6)	
Missing	61			
**Total number of children**				
0	1532 (27.8)	89 (5.8)	1443 (94.2)	<0.001
1-4	2279 (41.4)	397 (17.4)	1882 (82.6)	
5+	1700 (30.8)	229 (13.5)	1471 (86.5)	
**Reproductive preferences**				
Using for spacing	3835 (69.6)	500 (13.0)	3335 (87)	0.001
Using for limiting	972 (17.6)	138 (14.2)	834 (85.8)	
Undecided	501 (9.1)	69 (13.8)	432 (86.2)	
Can’t get preg/NA	203 (3.7)	8 (3.9)	195 (96.1)	
**Occupation**				
Farming/Trading	4383 (79.5)	629 (14.4)	3754 (85.6)	<0.001
Civil/Public servant	79 (1.4)	21 (26.6)	58 (73.4)	
Student	1049 (19.1)	65 (6.2)	984 (93.8)	
**Owns functional phone**				
Yes seen	795 (14.4)	167 (21.0)	628 (78.9)	<0.001
Yes, not seen	714 (13.0)	111 (15.6)	603 (84.5)	
No	4002 (72.6)	437 (10.9)	3565 (89.1)	
**Wealth index** ^*^				
Poor	3495 (63.4)	396 (11.3)	3099 (88.7)	<0.001
All other quintiles	2016 (36.6)	319 (15.8)	1697 (84.2)	

*“Poor” represents poorest, poorer and poor quintile and “None Poor” represent the less poor and least poor quintile.

The median distance to a health facility was 1.5 kilometers (max: 5.32 kilometers) and approximately 87 percent of respondents live within 2 kilometers of a health facility. This is not surprising because of the proliferation of CHPS service points in the Upper East region ([20]).

The mean age of the respondents was 29.3 years (sd: 9,9 years) with about 66.4% of them less than 35 years old. About 66.1 per cent of the respondents were married with about one quarter of those married having a shared husband/partner (*note shown on table). Fertility is quite high with 41.4 percent of women having between 1–4 children and an additional 30.8 percent having 5+ children.

The table also suggests that women in this rural setting have embraced modern means of communication. More than a quarter (27.4 percent) of the participants reported having a functional mobile phone.

Table 1 also shows the association between each covariate and modern contraceptive use. It shows that exposure to integrated primary health care (measured by the distance between respondents residential compounds to the nearest health facility), the level of education attained, age, marital status, number of children born, ownership of a functional cell phone, the type of marital union and wealth index and reproductive preferences were all strongly associated with contraceptive use (P-value <0.001). Religion and place of residence were not associated with modern contraceptive use.

Overall, the prevalence of contraceptive use among the respondents was 13 percent. Contraceptive use among those women who live less than 2 kilometers of a health facility is 14.6 percent compared with 10.3 percent for women who live 2 kilometers or more from a health facility. This difference is statistically significant at 1% and presents preliminary evidence that distance to health facilities has an influence on contraceptive use.

Consistent with previous literature, educational attainment has an effect on contraceptive use. Among women who attained secondary/tertiary, 21.3 percent reported using modern contraception compared to 11.8 percent of those with only primary/JHS education and 12.9 percent of those with no formal schooling. As expected, contraceptive use varies with age. Only 5.6 percent of 15–19 year olds used modern contraceptives, compared with about 13 percent of 35–49 year olds and 17 percent of 20–34 year olds. A little higher proportion of urban dwellers were contraceptive users compared to rural dwellers (14.1 percent vs 12.8 percent: p < 0.05). Religious affiliation was not statistically associated with contraceptive use.

Marital status is also a significant determinant of contraceptive use. Married women in monogamous relationships were more than twice likely to use modern contraceptives than single women (18.1 percent vs 7.4 percent). Similarly, women with a shared husband/partner were less likely to use contraception compared to those in monogamous marital unions (11.8 percent versus 18.1 percent: p < 0.001).

Most women who use modern contraception (about 70 percent) in this setting do so for purposes of spacing births. Among women who do not want to give birth, only 19 percent reported using contraception at the time of the survey, suggesting that there is large unmet need for family planning. However, we realized that 3.9 per cent of women who reported that they could not get pregnant reported using modern contraceptives which may suggest that such women are using contraceptives for reasons other than for preventing pregnancy. It is very likely that such women may be using contraceptives (barrier methods) to avoid getting infected with STIs/STDs.

Contraceptive use is also related to the number of children a woman has already had. Contraceptive use was highest among women who had between 1–4 children (17.4 percent), followed by those with 5 or more children (13.5 percent). Women who did not have a child were least likely to use contraceptives. Only 5.8 percent of these women reported being on contraception at the time of the survey.

A higher fraction of women (27 percent) who reported being public/civil servants were contraceptive users compared to those who were farmers or traders (14.4 percent). About 6 percent of students’ respondents were using contraceptives at the time of the survey. About 16 percent of those in the “poor” SES category reported being on contraception compared to 11 percent of those in the “other wealth index”.

Ownership of a functional mobile phone was strongly associated with contraceptive use. Twenty one percent of women who owned a functional mobile phone; (inspected by the data collectors); were on contraceptives at the time of the survey compared with only 10 percent of those who did not have a functional mobile phone (p < 0.001). An additional 15 percent of those who owned a functional mobile phone but which was not inspected by the data collectors; were also on modern contraceptives.

**Figure 2:**
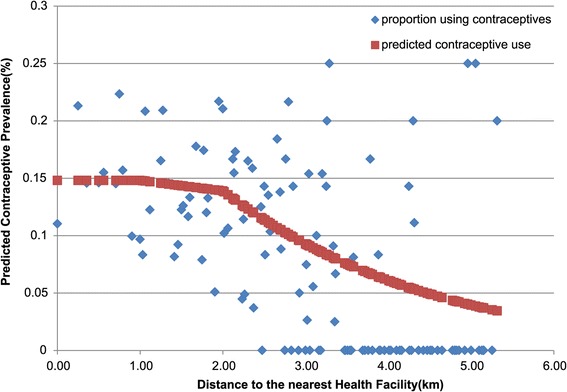
**Graph of restricted quadratic spline modeling distance in kilometers and contraceptive use by respondents.**

Figure 2 shows a quadratic splines modeling distance to the nearest health facility. The graph shows the sudden falloff in modern contraceptive use among women who reside beyond 2 km distance from a health facility. As such, we categorized our distance variable into those who reside within 2kilometers of a CHPS compound and those who reside beyond 2 kilometers. Owing to the clustered nature of settlements in this setting, 87 percent of all study participants live within a 2 kilometer reach of a health facility. About 15 percent of these residents use modern contraception. In contrast, a lower proportion (10.3) percent of women who live 2 kilometers or further away from the nearest health facility use modern contraception.

We employed multivariate logistic regression analysis to estimate the independent effect of exposure to the nearest health facility on contraceptive use among women, controlling for the effects of other covariates such as education, mothers age, marital status, and ownership of functional mobile phone, type of marriage, wealth index, parity and reproductive preferences. Table 2 presents the results from the multivariate regression analysis. Significant determinants of modern contraceptive use include exposure to the nearest health facility (measured as the distance between respondents residence to the nearest health facility), having attained Senior High School or higher education, fertility, possession of a functional cell phone, being in a monogamous union, and belonging to the lower wealth quintile.

**Table 2:** **Determinants of modern contraceptives use among rural women of reproductive age in the Upper East Region of Ghana**

	**Contraceptive use among 15–45 year old women in UER**			
**Variable**	**N = 5511**	**Unadjusted odds ratio 95% CI**	**Adjusted odds ratio 95% CI**	**P = value**
**Exposure to nearest health facility**				
<2 km	4796 (87.0)	1	1	
> = 2 km	715 (13.0)	0.67 (0.51, 0.88)	0.74 (0.57, 0.98)	0.035
**Level of education attained**				
None	3352 (60.8)	1	1	
Primary/JHS	1877 (34.1)	0.90 (0.74, 1.11)	1.28 (0.98, 1.66)	0.069
SHS/Tertiary	282 (5.1)	1.83 (1.35, 2.47)	1.76 (1.24, 2.50)	0.002
**Mother’s age**				
15-19	1211 (21.9)	1	1	
20-34	2449 (44.5)	3.39 (2.58, 4.49)	1.41 (0.89, 2.21)	0.137
35- 49	1851 (33.6)	2.42 (1.77, 3.31)	1.07 (0.65, 1.76)	0.799
**Marital status**				
Not married	1804 (32.7)	1	1	
Married/polygamous union	1374 (24.9)	1.65 (1.25, 2.20)	1.02 (0.72, 1.43)	0.923
Married/monogamous union	2272 (41.2)	2.74 (2.17, 3.45)	1.56 (1.17, 2.07)	0.003
**Owned functional mobile phone**				
No	4002 (72.6)	1	1	
Yes, Seen	795 (14.4)	2.15 (1.72, 2.68)	1.67 (1.31, 2.13)	<0.001
Yes, not seen	714 (13.0)	1.49 (1.16, 1.90)	1.20 (0.91, 1.58)	0.185
**Place of residence**				
Urban	716 (13.0)	1		
Rural	4795 (87.0)	0.89 (0.69, 1.15)	*	
**Religious Affiliation**				
Christianity	3073 (55.8)	1		
Traditional	728 (13.2)	0.97 (0.79, 1.19)	*	
Islam	1508 (27.4)	0.84 (0.64, 1.09)	*	
No Religion	202 (3.6)	0.73 (0.39, 1.37)	*	
Wealth index**				
Poor	3495 (63.4)	1	1	
None poor	2016 (36.6)	1.46 (1.21, 1.76)	1.31 (1.08, 1.58)	0.007
**Parity**				
0	1532 (27.8)	1	1	
1-4	2279 (41.4)	3.41 (2.58, 4.51)	2.62 (1.67, 4.07)	<0.001
5+	1700 (30.8)	2.51 (1.89, 3.34)	3.01 (1.89, 4.80)	<0.001
**Reproductive preferences**				
Using for spacing	3835 (69.6)	1	1	
Using for limiting	972 (17.6)	3.64 (1.33, 9.94)	1.04 (0.84, 1.29)	0.678
Undecided	501 (9.1)	4.03 (1.45, 11.19)	1.37 (0.93, 2.02)	0.114
Can’t get preg/NA	203 (3.7)	3.89 (1.39, 10.89)	0.32 (0.11, 0.91)	0.033

*Not statistically significant at 5 percent level of significance.

**“Poor” represents the Poorest, Poorer and Poor wealth quintiles and “None Poor” represents the Less Poor and Least poor wealth quintile.

The results show that women who live 2 km or more away from the nearest CHPS compound were less likely to use contraceptive compared to those who live within 2 km of a CHPS compound. As observed at the bivariate level, contraceptive use increases with increasing level of education after controlling for other variables. The odds of contraceptive use among women who have had a primary/JSS education is 1.28 times higher relative to women who have no education; [OR = 1.28] 95 CI (0.98, 1.66)], and those with senior high education or higher education were 1.76 times more likely to use modern contraceptives; [OR = 1.76] 95 CI (1.24, 2.50)].

The effects of the age of women on contraceptive use changed substantially in the multivariate analysis. The results indicate that the odds of contraceptive use decreases as a woman gets older. Even though 20–34 year old women were more likely to use modern contraception compared to 15–19 year olds, the difference was not statistically significant (OR = 1.41 [(p-value 0.137)]).

The type of marital union in this setting is associated with significantly higher contraceptive use. The odds of contraceptive use among women in monogamous relationships is 1.56 times compared to women who are not married and this result is statistically significant at a p-value = 0.003. There are no significant differences between women in polygamous unions and single women.

Ownership of a functional mobile phone is also highly associated with contraceptive use. The odds of contraceptive use among women with a functional mobile phone which is seen by an interviewer is 1.67 times compared to women who don’t have a mobile phone (p-value = <0.001). There was however no significant difference between women who reported that they own a phone although the device was not seen and those who reported that they did not own a phone.

Wealth index was statistically associated with contraceptive use even after controlling for all the other covariates. Women in the “None poor” households are 31 percent more likely to use modern contraceptive than those in the “Poor” wealth index [OR = 1.31] -value = 0.007)].

The results also reveal strong association between parity and modern contraceptive use with higher parity associated with a higher likelihood of contraceptive use. The odds of contraceptive use among women who have 1–4 children is 2.62 times compared to women with no children. The odds of contraceptive use among women who had 5 or more children, was threefold the odds of use among women who had no child [OR = 3.01] 95 CI (1.89, 4.80)].

## Discussion and conclusion

This study assessed the effect of proximity to service points relative to the effects of social determinants of contraceptive use. Our findings provide evidence that proximity to functional health facility; largely CHPS compound is strongly associated with modern contraceptive use. We found that the contraceptive prevalence rate was reasonably uniform for women who lived within 2 km of a service point, but use falls off monotonically for distances greater than 2 km. Our findings lend support to earlier research that examined the relationship between service availability and contraceptive use in rural Guatemala. Using DHS data for cluster analysis, Thomas W. Pullum found that distance and travel time to facilities remained critically important even after controlling for socioeconomic factors [21].

However, our results also portrayed a non-lineal relationship between distance to the nearest health facility and contraceptive use suggesting that women do not necessarily have their contraceptives services at the nearest facility to their residence. We suspect this may be attributed to the quality of services, attitude of service providers towards clients and fear of stigmatization or domestic violence resulting from use of contraceptives especially in relationships where male partners disapprove of their wives use of contraceptives [21-23]. For instance, RamaRao and colleagues found that contraceptive use increased steadily with quality, with predicted probabilities of contraceptive use being 55%, 62% and 67% for low-quality care, medium-quality care and high-quality care respectively. In contrast, Ngom and colleagues noted deep seated community “gatekeeping” over women’s health-seeking behavior and concluded that when women and children become ill in profoundly gender-stratified societies like those of northern Ghana, they are often denied the timely provision of simple, life-saving interventions because their elder women or male relatives are reluctant to allow them to seek care immediately. This problem is particularly constraining for family planning services in Northern Ghana and other parts of Africa and induces clandestine use of contraceptives [12].

The findings also highlight the importance of individual and household level characteristics that influences contraceptive use such as level of education attained, marital status and the type of marriage, ownership of a functional mobile phone, the level of socioeconomic status, couple preference, and parity.

Our results showed a strong association between formal education and contraceptive use among women. This is however not surprising because considerable amount of evidence abound in Africa and elsewhere on the powerful effects of formal education on female empowerment, reproductive health, child and maternal health. Formal education is particularly noted to be a strong predictor of contraceptive use [24-26]. Cleland and colleagues (1988) noted for instance that on average, each one-year increment in mother’s education corresponds to a 7–9% decline in under-5 s mortality. Yet in this setting, female formal education remains very low. Only 5% of the women in this study have had secondary or tertiary education and as high as 60.8% of the women have never been to school. The low level of female education reflects generally low levels of education in the region reinforced by patriarchal values systems that marginalizes female education and empowerment.

Compared to single women, the odds of modern contraceptive use among married women was almost doubled the odds of use among single women and this was highly significant at 5 percent confidence level. This was however expected as several studies have consistently shown a positive correlation between marriage and modern contraceptive use. According to the 2008 Ghana DHS report, contraceptive prevalence rate increased from 22 percent among currently married women in 1998 to 25 percent in 2003, but declined in the past five years—24 percent in 2008—a reversal in the trend [17]. It is instructive to note that women in monogamous relationships in this setting are more likely to use contraception than those who share a husband/partner. It appears in polygynous relationships in this and similar settings in Africa, there is a rotational system of wives sexual gate-keeping roles over their husband. For instance, Achana et al. [24] observed that women perceptions of a partner’s risky sexual behavior influence their contraceptive use including condom use and even sexual refusal. Women in such unions often adjust to an informal rotation system where breastfeeding mothers lose the attention of their husbands to their rivals; imposing a responsibility on the wife whose turn it is to serve and keep watch over their husband; and this may lead to low risk perceptions and failure to use contraceptive.

Our results also revealed a strong association between ownership of a functional mobile phone and use of contraception among women. There has been rapid expansion of the communication industry in Ghana resulting in many people increasingly having access to mobile phones. According to the 2010 Population and Housing Census, close to half (47.7 percent) of persons 12 years and older in the country and 24.2 percent in the Upper East Region respectively owned a mobile phone [14]. Perhaps ownership of mobile phone might be a reflection of better socio-economic status which explains women’s ability to afford contraceptives. An additional explanation for the strong association between ownership of a mobile phone and contraceptive use might be the fact that mobile phones are channels for effective health communication and promotion of behavior change [21-23] Recently, Walakira and colleagues [27] in a longitudinal trial in the Jinja District in the Eastern region of Uganda found out that text messaging had a profound impact on family planning service uptake. Nearly 39 percent and 14 percent of women in the implementation and control groups respectively started using a modern contraceptive method after the intervention, with most women opting to use the injectable. Thirteen percent of the women in the implementation group chose to use long acting methods of contraceptives compared to none of the women in the control group. Indeed, recent reviews [28,29] suggest that the use of text messages can be used successfully to promote behaviour change in smoking, dieting, and physical activity.

Our findings have serious programmatic and policy implications not only for the Upper East Region but Ghana overall. When effectively implemented, CHPS has been shown to have profound effects on fertility and child mortality [13]. Yet, CHPS roll out has been very slow in most parts of Ghana [13]. At the present rate of CHPS roll out, the country’s aim of scaling up CHPS to all communities by 2015 will not be attainable with retrogressive implications for child and maternal health.

Our study has a couple of limitations. For instance, the data is from a cross-sectional survey and so suffers temporal defects. We are unable to establish any causal relationship between our outcome of interest (contraceptive use) and the covariates of interest. We were also limited by the fact that the survey did not collect data on the supply side of contraception. We are therefore unable to determine the extent to which supply factors such as stock-outs, distribution mechanisms and the effects of changes in policy programs may have influenced contraceptive use or non-use in the study setting.

While we cannot estimate the extent to which these factors may have affected our results, we strongly believe that rapid scale up of functional CHPS accompanied with improved access to health services which address client’s needs would kindle uptake of modern contraceptive use, reduce unwanted pregnancies and hasten the attainment of MDG 4 & 5 in Ghana. Achieving this will require stronger political will, effective leadership at the District and Sub-district level, qualitative training and deployment of competent Community Health Officers and behavioural and communication interventions that addresses the needs of clients.

## Abbreviations

GEHIP: Ghana essential health intervention project
UER: Upper east region
CHPS: Community-based health planning and services
TBAs: Traditional birth attendants
EAs: Enumeration areas
IRB: Institutional review board

## References

[1] Ross J, Hardee K (2013). Access to contraceptive methods and prevalence of use. J Biosoc Sci.

[2] Awoonor-Williams JK, Sory EK, Nyonator FK, Phillips JF, Wang C, Schmitt ML (2013). Lessons learned from scaling up a community-based health program in the Upper East Region of northern Ghana. Glob Health Sci Pract.

[3] Nyonator FK, Awoonor-Williams JK, Phillips JF, Jones TC, Miller RA (2005). The Ghana community-based health planning and services initiative for scaling up service delivery innovation. Health Policy Plan.

[4] Awoonor-Williams JK, Vaughan-Smith MN, Phillips JF, Malarcher S (2010). Scaling-up health system innovations at the community level: A case study of the Ghana experience. Social Determinants of Sexual and Reproductive Health: Informing Future Research and Programme Implementation.

[5] Services GS, Services GH, Macro I (2008). Ghana Demographic and Health Survey.

[6] Asamoah BO, Agardh A, Ostergren P-O (2013). Inequality in fertility rate and modern contraceptive use among Ghanaian women from 1988–2008. Int J Equity Health.

[7] Adongo PB, Phillips JF, Kajihara B, Fayorsey C, Debpuur C, Binka FN (1997). Cultural factors constraining the introduction of family planning among the Kassena-Nankana of northern Ghana. Soc Sci Med.

[8] Avogo W, Agadjanian V (2008). Men’s social networks and contraception in Ghana. J Biosoc Sci.

[9] Bongaarts J, Bruce J (1995). The causes of unmet need for contraception and the social content of services. Stud Fam Plann.

[10] Ezeh AC (1993). The influence of spouses over each other’s contraceptive attitudes in Ghana. Stud Fam Plann.

[11] Bawah AA (2002). Spousal communication and family planning behavior in Navrongo: a longitudinal assessment. Stud Fam Plann.

[12] Bawah A, Akweongo P, Simmons R, Phillips JF (1999). Women’s fears and Men’s anxieties: the impact of family planning on gender relations in northern Ghana. Stud Fam Plann.

[13] Awoonor-Williams JK, Bawah AA, Nyonator FK, Rofina A, Oduro A, Ofosu A (2013). The Ghana essential health interventions program: a plausibility trial of the impact of health systems strengthening on maternal & child survival. BMC Health Serv Res.

[14] Ghana Statistical Service (2012). 2010 Population and Housing Census Final Results.

[15] Nyonator FK, Awoonor-Williams JK, Phillips JF (2011). Scaling Down to Scale-up: Accelerating the Expansion of Coverage of Community-based Health Services in Ghana.

[16] Annual Report-UER. Annual health sector report, Bolgatanga, 2010. Upper East Regional Health Administration, Ghana Health Service.

[17] Ghana Statistical Service (2009). Ghana Demographic and Health Survey 2008.

[18] Stephenson R, Baschieri A, Clements S, Hennink M, Madise N (2007). Contextual influences on modern contraceptive use in sub-Saharan Africa. Am J Public Health.

[19] Hosmer DW, Lemeshow S (2000). Applied Logistic Regression.

[20] Awoonor-Williams JK, Elias Kavinah S, Nyonator FK, Phillips JF, Chen W, Schmitt ML (2013). Lessons learned from scaling up a community-based health program in the Upper East Region of northern Ghana. Global Health: Sci. &Pract.

[21] Pullum TW (1991). The Relationship of Service Availability to Contraceptive Use in Rural Gauatemala. Demographic and Health Surveys, Institute for Resource Development.

[22] RamaRao S, Lacuesta M, Costello M, Pangolibay B, Jones H (2003). The link between quality of care and contraceptive use. Int Fam Plan Perspect.

[23] Ngom P, Debpuur C, Akweongo P, Adongo P, Binka FN (2003). Gate-keeping and women’s health seeking behaviour in Navrongo, northern Ghana. Afr J Reprod Health.

[24] Achana SF, Akweongo P, Debpuur C, Cleland J (2009). Coping strategies of young mothers at risk of HIV/AIDS in the Kassena-Nankana district of Northern Ghana. Afr J Reprod Health.

[25] Maharaj P, Cleland J (2014). Marital and Cohabiting Condom Use Within in KwaZulu-Natal, South Africa Partnerships.

[26] Cleland JC, Ginneken KVAN, Jo SI (1988). Maternal education and child survival in developing countries : the search for pathways of influence. Soc Sci Med.

[27] Walakira B, Lubaale YAM, Balidawa F, Nalule S, Githinji F. Can Mobile Phone Text Messaging Increase Uptake of Family Planning Services in Uganda? MEASURE Evaluation PRH Working Paper Series. March 2013. WP-13-135.

[28] Cole-Lewis H, Kershaw T (2010). Text messaging as a tool for behavior change in disease prevention and management. Epidemiol Rev.

[29] Fjeldsoe BS, Marshall AL, Miller YD (2009). Behavior change interventions delivered by mobile telephone short-message service. Am J Prev Med.

